# Progression of Chronic UVB-Induced Skin Damage During Long-Term Topical Nicotinamide Mononucleotide Treatment in Mice

**DOI:** 10.3390/ijms27219807

**Published:** 2026-10-31

**Authors:** Pawit Phadungsaksawasdi, Kanyawan Harnsiriwattana, Kodchakorn Chukanchitipat, Napat Hunsajarupan, Adiluck Pisutpunya

**Affiliations:** 1 Division of Dermatology, Chulabhorn International College of Medicine, Thammasat University, Khlong Luang 12120, Pathum Thani, Thailand; 2 Department of Pathology, Faculty of Medicine, Thammasat University, Khlong Luang 12120, Pathum Thani, Thailand; adiluckp@tu.ac.th

**Keywords:** nicotinamide mononucleotide, chronic UVB exposure, photodamage, epidermal dysplasia

## Abstract

Nicotinamide mononucleotide (NMN), a precursor of nicotinamide adenine dinucleotide (NAD^+^), is widely investigated as a potential anti-aging intervention. However, its long-term efficacy and safety, particularly in topical formulations applied to chronically photodamaged skin, remain unclear. This study evaluated whether topical 5% NMN modifies the progression of chronic UVB-induced skin damage in mice. Female C57BL/6NJcl mice were assigned to four groups: non-irradiated control, UVB alone, UVB plus vehicle cream, and UVB plus 5% NMN cream. UVB was delivered to shaved dorsal skin three times weekly at 120 mJ/cm^2^, and cream was applied after each irradiation through week 25. UVB-exposed groups developed progressive skin damage, with the UVB plus NMN group showing a nonsignificant trend toward greater clinical severity at week 25 (*p* = 0.08). Histological analysis showed significant increases in epidermal thickness and dysplasia from week 12 to the late endpoint only in the UVB plus NMN group. These changes were accompanied by marked induction of *Krt6*, *Krt16*, and *S100a8*, whereas *Krt1* showed no significant change. In contrast, no increase in *Parp1* and *Sirt1* was observed at the transcriptional level in the NMN group. Parp1 and Sirt1 were significantly upregulated in the UVB alone and UVB plus vehicle groups but not in UVB plus NMN-treated skin. Overall, these findings indicate that long-term topical NMN did not protect chronically UVB-exposed mouse skin and may have contributed to the worsening of photodamage.

## 1. Introduction

Nicotinamide mononucleotide (NMN) is a key biosynthetic precursor of nicotinamide adenine dinucleotide (NAD^+^), a central coenzyme involved in metabolic regulation, DNA repair, and cellular stress responses [1,2,3]. Through NAD^+^ replenishment, NMN has attracted considerable interest as an anti-aging compound and as a potential therapeutic agent for age-related and degenerative conditions. In dermatology-related models, systemic NMN administration has shown protective effects against acute and short-term ultraviolet B (UVB)-induced skin damage in mice, including preservation of epidermal architecture, modulation of inflammatory pathways, and enhancement of antioxidant defenses [4,5,6]. These findings have stimulated interest in the potential of the NMN/NAD^+^ pathway to prevent or reduce cellular photodamage.

UVB exposure damages the epidermis through interconnected mechanisms involving DNA damage, oxidative stress, inflammation, and dysregulated keratinocyte proliferation. UVB directly induces DNA photolesions, particularly cyclobutane pyrimidine dimers, while also increasing reactive oxygen species that further contribute to oxidative DNA damage [7,8]. These cellular stresses promote inflammatory responses and disrupt epidermal homeostasis [7,8]. With repeated or prolonged exposure, persistent DNA damage and abnormal keratinocyte proliferation may contribute to epidermal hyperplasia, dysplasia, photoaging, and ultimately photocarcinogenesis [7,8,9].

NAD^+^-dependent enzymes provide a plausible mechanism for these protective effects. Sirtuins, including SIRT1, are NAD^+^-dependent deacetylases involved in genomic maintenance, mitochondrial function, oxidative stress defense, and inflammatory regulation [1,2,3]. PARPs, including PARP1, also consume NAD^+^ during DNA-damage detection, repair, and chromatin remodeling [1,10]. Restoration of NAD^+^ availability may therefore support DNA repair and cellular homeostasis after UV-induced injury [1,2]. However, emerging evidence suggests that NAD^+^ metabolism and sirtuin activity may have context-dependent effects. Although NAD^+^ replenishment may protect normal cells from acute stress, enhanced metabolic capacity and repair signaling could also support the persistence or expansion of abnormal keratinocytes in chronically damaged or premalignant tissue [3,11,12].

Although NMN has shown beneficial effects in acute models of skin injury, its long-term effects under chronic UV stress remain unclear. Chronic UVB exposure causes cumulative epidermal damage, persistent inflammation, altered differentiation, and premalignant keratinocyte changes [13,14]. In UV-exposed mice, oral NMN increased skin NAD^+^ levels but did not reduce tumor development [15], while another study reported increased proliferation and tumor aggressiveness following NMN supplementation [16]. In humans, oral nicotinamide (NAM) has demonstrated protective effects against UV-induced damage and reduced keratinocyte cancers and actinic keratoses in high-risk immunocompetent individuals [17], whereas no reduction in keratinocyte cancers was observed in solid-organ transplant recipients [18]. These findings suggest that the effects of NAD^+^-related interventions may depend on the biological context. However, NAM and NMN are distinct compounds and may not exert identical biological effects. Concerns have also been raised that sustained NAD^+^ augmentation may support the survival of damaged cells under conditions of persistent DNA damage [11,12], further highlighting the uncertain safety of NMN in chronically photodamaged skin. In this study, we evaluated topical 5% NMN in wild-type mice subjected to chronic UVB exposure using clinical, histological, and molecular assessments to provide insight into the safety and context-dependent effects of NMN in photodamaged skin.

## 2. Results

### 2.1. Body Weight Changes Remained Comparable Among Groups

**Figure 1:**
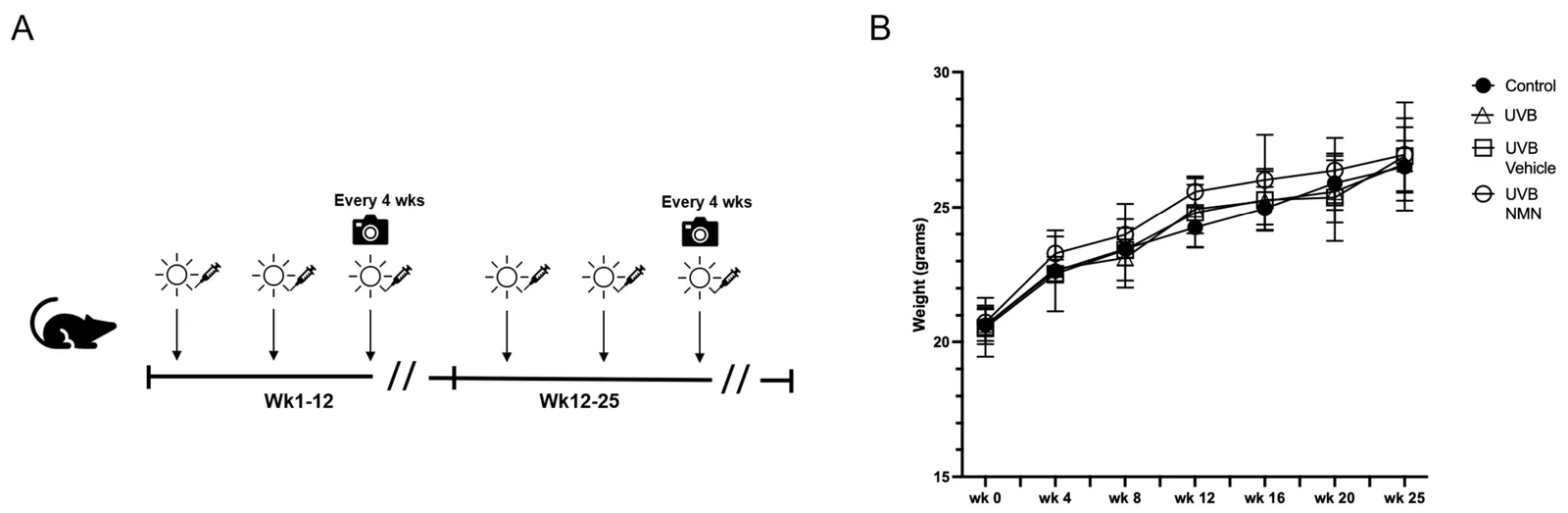
Experimental design and comparable body weight changes among groups: (**A**) Schematic overview of the 25-week UVB exposure and topical treatment protocol. (**B**) Body weight measured every 4 weeks increased progressively over time and remained comparable among the non-irradiated control, UVB alone, UVB plus vehicle, and UVB plus NMN groups (*n* = 6 per group).

Body weight increased progressively over time in all experimental groups and remained comparable among the control, UVB alone, UVB plus vehicle, and UVB plus NMN groups (Figure 1B). No group exhibited sustained weight loss or clear deviations from the overall physiological growth pattern. These findings suggest that the chronic UVB irradiation protocol and topical cream applications did not cause obvious growth impairment or systemic burden detectable by body weight monitoring.

### 2.2. Topical NMN Was Associated with Exacerbated Clinical Photodamage During Late Chronic Exposure

**Figure 2:**
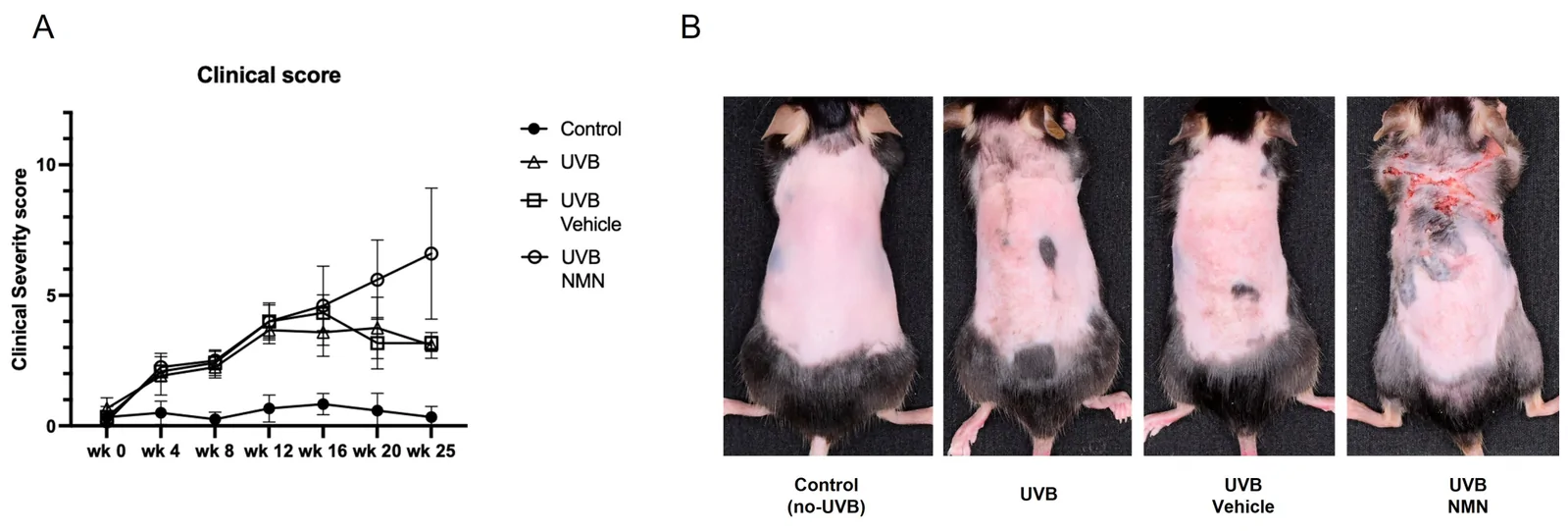
Clinical skin damage after chronic UVB exposure: (**A**) Serial clinical severity scores from week 0 to week 25. UVB-exposed groups developed progressive skin damage, with the UVB + NMN group showing a nonsignificant trend toward a higher mean score at the later time point (*n* = 6 per group). (**B**) Representative endpoint dorsal photographs showing differences in gross skin appearance among groups.

Clinical skin severity remained negligible in non-irradiated controls but increased progressively in all UVB-exposed groups (Figure 2). During the first half of UVB exposure, clinically detectable photodamage increased similarly in the UVB alone, UVB plus vehicle, and UVB plus NMN groups, indicating that topical NMN did not prevent clinically detectable UVB-induced skin damage during this period. From week 12 onward, however, the clinical scores of the UVB alone and UVB plus vehicle groups plateaued or slightly declined by the late endpoint. In contrast, clinical severity in the UVB plus NMN group continued to rise after week 16, resulting in a nonsignificant trend toward a higher mean severity score at week 25. Although this difference was not statistically significant, this late difference showed a trend toward higher severity in the UVB plus NMN group compared with the UVB alone group (*p* = 0.08).

### 2.3. Topical NMN Promoted Significant Epidermal Thickening and Histological Dysplasia After Prolonged UVB Exposure

**Figure 3:**
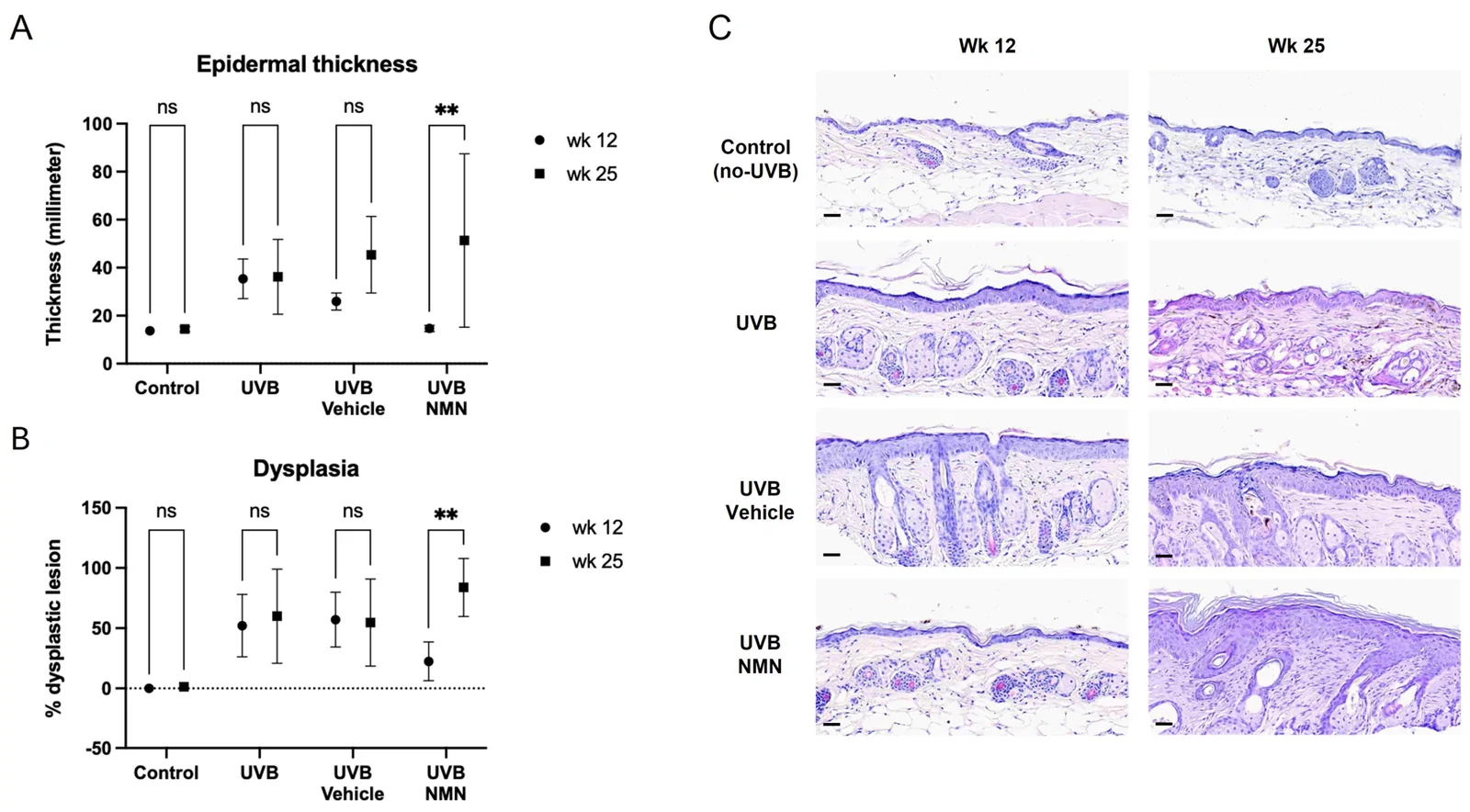
Epidermal thickening and histological dysplasia after chronic UVB exposure: (**A**) Epidermal thickness at week 12 and week 25. Significant within-group thickening from week 12 to week 25 was observed only in the UVB plus NMN group (*n* = 6 per group). (**B**) Percentage of dysplastic lesions at week 12 and week 25. Dysplastic lesions increased significantly over time only in the UVB plus NMN group. (**C**) Representative hematoxylin and eosin-stained dorsal skin sections showing histological changes at week 12 and week 25, with progressive epidermal thickening and architectural disorganization most evident in UVB plus NMN-treated skin at the late endpoint. Scale bar: 50 µm. ** *p* < 0.01; ns, not significant.

Histological evaluation revealed characteristic UVB-induced epidermal alterations across all irradiated groups, but the time-dependent progression and worsening of these lesions appeared more pronounced in the UVB plus NMN-treated skin (Figure 3). At week 12, both the UVB alone and UVB plus vehicle groups already displayed substantial epidermal thickening and early dysplastic changes, whereas histological changes in the UVB plus NMN group were only slight at this early time point. By week 25, however, this pattern reversed: epidermal thickness increased significantly from week 12 to week 25 exclusively in the UVB plus NMN group, whereas the control, UVB alone, and UVB plus vehicle groups did not show statistically significant within-group increases over this period (Figure 3A). Similarly, the proportion of dysplastic lesions increased significantly over time in the UVB plus NMN group (Figure 3B). Representative histological sections demonstrated epidermal morphological changes at weeks 12 and 25 (Figure 3C).

### 2.4. Topical NMN Induced a Hyperproliferative and Stress-Associated Epidermal Gene-Expression Profile

**Figure 4:**
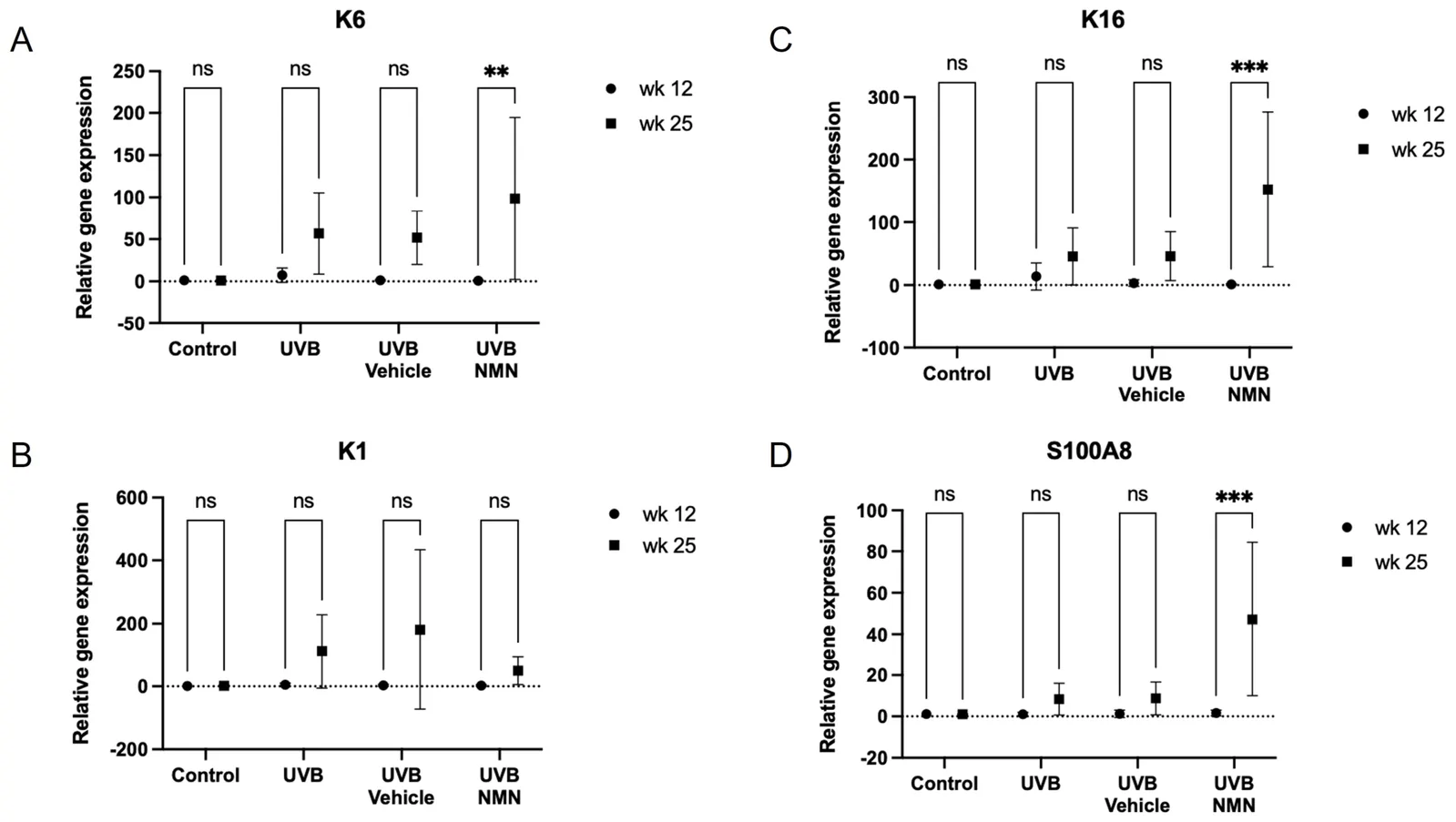
Expression of epidermal differentiation and hyperproliferation-associated genes: Relative mRNA expression of (**A**) *Krt6*, (**B**) *Krt1*, (**C**) *Krt16*, and (**D**) *S100a8* at week 12 and week 25. *Krt6*, *Krt16*, and *S100a8* increased significantly from week 12 to week 25 only in the UVB plus NMN group, whereas *Krt1* showed no significant within-group increase. ** *p* < 0.01; *** *p* < 0.001; ns, not significant.

To investigate the molecular mechanisms underlying the observed histological phenotypes, we analyzed the expression of markers associated with epidermal differentiation, injury response, and dysplasia (Figure 4). The expression of *Krt6*, a key stress-associated keratin, increased significantly from week 12 to the week 25 endpoint only in the UVB plus NMN group (*p* < 0.01), whereas no significant longitudinal changes were observed in the other groups (Figure 4A). A similar pattern was observed for *Krt16*, which demonstrated robust and significant induction in NMN-treated skin (*p* < 0.001; Figure 4C). The time course of these stress-associated keratin changes was consistent with the histological progression shown in Figure 3. Furthermore, *S100a8* was significantly upregulated over time in the UVB plus NMN group (*p* < 0.001; Figure 4D), suggesting a sustained inflammatory and abnormal epidermal response. In contrast, the differentiation marker *Krt1* showed no significant within-group increase and remained highly variable across the irradiated groups (Figure 4B). Together, these molecular findings indicate that chronic topical NMN treatment is associated with enhanced stress-associated keratin expression, epidermal hyperproliferation, and dysplasia, rather than protection against UVB-induced damage or promotion of normal terminal differentiation.

### 2.5. Topical NMN Was Associated with Reduced Transcriptional Upregulation of Parp1 and Sirt1 After Prolonged Chronic UVB Exposure

**Figure 5:**
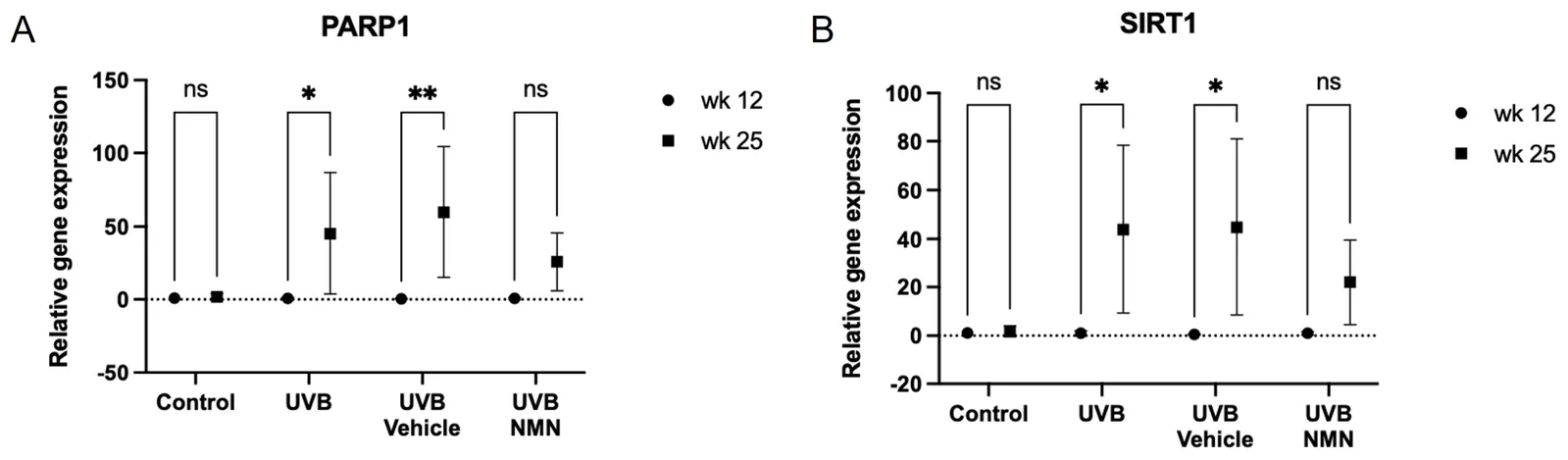
*Parp1* and *Sirt1* mRNA expression after chronic UVB exposure: Relative mRNA expression of (**A**) *Parp1* and (**B**) *Sirt1* at week 12 and week 25. *Parp1* and *Sirt1* were significantly upregulated from week 12 to week 25 in the UVB alone and UVB plus vehicle groups, whereas no significant longitudinal induction was observed in UVB plus NMN-treated skin. * *p* < 0.05; ** *p* < 0.01; ns, not significant.

PARP1 and SIRT1 are NAD^+^-dependent enzymes and play an important role in DNA repair regulation and cellular stress responses [1,10]. In this study, *Parp1* mRNA levels increased significantly from week 12 to the week 25 endpoint in both the UVB alone (*p* < 0.05) and UVB plus vehicle groups (*p* < 0.01), whereas no significant longitudinal changes were observed in the non-irradiated control or UVB plus NMN groups (Figure 5A). A similar pattern was observed for *Sirt1*, which was significantly upregulated over time in the UVB alone and UVB plus vehicle groups (*p* < 0.05), but showed no significant longitudinal increase in the UVB plus NMN group (Figure 5B).

## 3. Discussion

A protective effect of long-term topical NMN against chronic UVB-induced skin damage was not demonstrated in this study. Over the 25-week exposure period, the UVB plus NMN group showed a nonsignificant trend toward greater clinical severity at the endpoint, significant longitudinal increases in epidermal thickness and dysplasia, and upregulation of *Krt6*, *Krt16*, and *S100a8*. No significant longitudinal increases in *Parp1* and *Sirt1* were detected in the UVB plus NMN group, whereas increases were detected in the UVB-alone and UVB plus vehicle groups. Together, these findings document progression of UVB-induced skin damage during topical NMN treatment. The mechanisms underlying these observations remain unresolved because cutaneous NAD^+^ availability and related metabolites were not measured.

The histological and molecular changes are consistent with a chronic maladaptive response under sustained UVB exposure. However, the nonsignificant clinical severity difference and within-group analyses do not establish that NMN caused harm or altered progression relative to the other treatment groups. *Krt6*, *Krt16*, and *S100a8* were upregulated in the NMN-treated group, accompanied by histological changes consistent with chronic hyperproliferative, inflammatory stress, and dysplasia-related responses [19,20]. The lack of *Krt1* upregulation suggests limited restoration of normal terminal keratinocyte differentiation. In addition, PARP1 is an NAD^+^-dependent enzyme involved in DNA-damage sensing, chromatin remodeling, and DNA repair regulation, whereas SIRT1 is a major NAD^+^-dependent deacetylase that contributes to cellular stress adaptation and genomic stability [1,2,10]. Interestingly, no statistically significant longitudinal increases in *Parp1* and *Sirt1* mRNA were detected in the NMN-treated group. One possible explanation is that the treatment may have altered feedback regulation, or that the extent of genotoxic injury limited the capacity of the transcriptional stress response. However, mRNA levels do not establish protein abundance or enzyme activity; these mechanisms remain speculative and require further investigation.

Our results contrast with some previous studies reporting protective effects of NMN against ultraviolet-induced skin injury in murine models [4,5,6]; however, these studies differ in biological context and route of administration. The effects of NAD^+^-related interventions remain controversial in the literature and may vary depending on the biological context. This complexity is further supported by studies of related vitamin B3 derivatives. In vitro studies have shown that nicotinamide exerts protective effects against UVB-induced damage in keratinocytes [21,22], whereas in vivo studies have reported that oral NMN did not protect against UV-induced skin carcinogenesis in mice [15] and may potentially promote skin tumor development under certain conditions [16]. In humans, oral nicotinamide reduced the incidence of keratinocyte cancers in high-risk immunocompetent patients [17] but did not demonstrate a significant benefit in immunosuppressed solid-organ transplant recipients [18]. In addition, whole-blood NAD^+^ levels may not accurately reflect tissue-specific NAD^+^ dynamics or local biological responses [23]. In our in vivo study, topical NMN was applied to chronically UVB-damaged skin, a setting that differs from short-term UV exposure and systemic administration. In this context, NMN may support repair in normal keratinocytes while also promoting the survival of damaged cells. Together, these findings suggest that the effects of NAD^+^-related interventions are context dependent and may vary according to the route of administration, timing, and presence of pre-existing skin damage, while the mechanisms underlying their long-term effects require further investigation.

The established role of NMN as an NAD^+^ precursor provides a biological rationale for considering NAD^+^-dependent mechanisms [3]. However, the present study did not determine whether topical NMN increased cutaneous NAD^+^ availability. One possible explanation is that increased NAD^+^ availability, if achieved, could support the persistence of damaged keratinocytes under chronic genotoxic stress. Although NAD^+^ is essential for DNA repair and cellular recovery, it could also support the metabolic requirements and survival of cells harboring unrepaired DNA damage [11,12]. A second possibility is that NMN alters the local immune microenvironment, thereby influencing the clearance or persistence of damaged cells and disrupting normal cutaneous homeostasis [24]. These mechanisms remain untested, and the findings cannot be attributed specifically to NAD^+^ replenishment. A third possibility is a direct cutaneous effect of topical NMN, including irritation or hypersensitivity. Without an NMN-only group, such effects cannot be excluded, and the safety of topical 5% NMN on non-irradiated skin remains unresolved. Although systemic NMN has shown favorable tolerability in both short- and long-term in vivo studies [25] and short-term human trials [26], and its precursor nicotinamide has an established safety profile in topical formulations [27], evidence regarding the long-term safety of topical NMN remains lacking in both in vivo models and humans.

There are several limitations to this study. First, the experimental sample size was relatively small. Second, the study did not include an NMN-only group without UVB exposure; therefore, the potential toxicity of NMN itself could not be determined. Third, different concentrations of NMN were not evaluated, and a dose–response relationship was not assessed. Fourth, the use of a single mouse strain limits the generalizability of our findings, as skin characteristics and responses to UVB may differ across mouse strains and sexes. In addition, differences between murine and human skin limit the direct extrapolation of these findings to humans. Finally, tissue NAD^+^ levels and related enzymatic activities were not measured, which limits the mechanistic interpretation of the findings.

## 4. Materials and Methods

### 4.1. Animals and Study Design

Wild-type mice (C57BL/6NJcl) were purchased from Nomura Siam International (Thailand). Seven- to eight-week-old female wild-type mice were used in this study. Female mice were selected to minimize aggression-related injuries during the experiment. All procedures were performed in accordance with the guidelines for proper conduct of animal experiments and the protocols approved by the Thammasat University Institutional Animal Care and Use Committee (IACUC) for Animal Experiments Protocol Number 022/2022, 005/2025. Nicotinamide mononucleotide was purchased from Winkey Technology Co Ltd. (Shenzhen, China). The vehicle cream and 5%NMN cream were prepared by Skin Intimate Co., Ltd. (Nonthaburi, Thailand), with the formulation provided in the Supplementary Materials. The 5% concentration was selected based on a previous study using 5% Nicotinamide for UV-related skin effects [28]. The NMN cream and vehicle cream were stored at 4–8 °C and protected from light. Dorsal hair was trimmed biweekly using a standard laboratory clipper. Hair removal cream (Veet) was used three times during the experimental period to prepare the skin and remove excess hair.

Mice were assigned to four groups (*n* = 6 per group): non-irradiated control, UVB alone, UVB plus vehicle cream, and UVB plus 5% NMN cream. Broadband UVB lamps (Philips TL 40W/12 RS, Warsaw, Poland) were used. The dorsal skin was irradiated with UVB at an irradiance of 0.5 mW/cm^2^ for 4 min, delivering a fluence of 120 mJ/cm^2^. This dose was selected as a sub-minimal erythema dose based on preliminary MED testing in C57BL/6NJcl mice using the same irradiation setup. UVB irradiation was performed three times per week for up to 25 weeks. For topical treatment groups, vehicle or 5% NMN cream (pea-sized amount) was applied to the dorsal skin within 20 min after UVB irradiation. Post-irradiation application was chosen to assess skin repair following UVB exposure and to avoid potential UV absorption or reflection by the cream during irradiation. The interval was selected for practical reasons and to standardize treatment timing across sessions. Body weight was recorded every 4 weeks. Mice were euthanized at the end of the experiment at week 25 (Figure 1A).

### 4.2. Clinical Severity Score

Skin changes in each group were assessed biweekly. Each picture was evaluated using skin severity scores by trained persons. The scoring criteria were composed of erythema, edema, and excoriation/erosion, with grading from 0–3; 0 = none, 3 = severe. The average score for each group of mice was calculated from individual scores.

### 4.3. Histology Assessment

Dorsal skin collected at weeks 12 and 25 was fixed in formalin, embedded in paraffin, sectioned at 4 µm thickness, and stained with hematoxylin and eosin (H&E). The H&E slides were digitized using a whole-slide imaging (WSI) system. Epidermal thickness and the proportion of dysplastic lesions were blindly evaluated by a pathologist using the digital whole-slide images.

### 4.4. Gene-Expression Analysis

Total RNA was extracted from snap-frozen dorsal skin samples collected at weeks 12 and 25 using RiboEx (GeneAll, Seoul, Republic of Korea) according to the manufacturer’s instructions. cDNA was synthesized from total RNA using iScript reagents (Bio-Rad, Hercules, CA, USA). The mRNA expression was analyzed by qPCR using Luna Universal qPCR Master Mix (New England Biolabs, Ipswich, MA, USA). The primer sequences used for qPCR are listed below. Primers were designed using NCBI Primer-BLAST to be about 19–22 bp in length, with a GC content of 40–60% and a melting temperature (Tm) of about 60 °C (Table 1). The PCR product sizes ranged from 70 to 200 bp. Secondary structures and dimer formation were controlled with Amplify4 software. Primer specificity was evaluated in silico against the mouse reference genome (GRCm39). The reaction conditions were as follows: 95 °C for 180 s, followed by 40 cycles of 95 °C for 10 s, 60 °C for 30 s, and 72 °C for 30 s. A melting curve was generated at the end of each PCR to verify specific amplification of the target cDNA. The negative control contained no DNA template. Relative transcript levels were calculated using the threshold cycle (2-ΔΔCT) method and normalized to GAPDH mRNA levels.

### 4.5. Statistical Analysis

Longitudinal clinical scores were analyzed using a mixed-effects model followed by Tukey’s multiple comparisons test. Data comparing week 12 and the late endpoint (week 25) were analyzed using repeated-measures two-way ANOVA followed by Šídák’s multiple comparisons test to compare time points within each group. Statistical significance was set at *p* < 0.05. All statistical analyses were performed using GraphPad Prism (version 9.0).

## 5. Conclusions

In conclusion, a protective effect of topical 5% NMN against chronic UVB-induced skin damage was not demonstrated over the 25-week study period. In the UVB plus NMN group, epidermal thickness and histological dysplasia increased significantly between weeks 12 and 25. These changes were accompanied by robust induction of stress- and inflammation-associated markers, including *Krt6*, *Krt16*, and *S100a8*, without an increase in the transcriptional levels of the NAD^+^-dependent, repair-associated genes *Parp1* and *Sirt1*. These findings suggest that the effects of NMN may depend on biological context, exposure duration, and route of administration. Whether these responses involve altered NAD^+^ metabolism remains unknown and requires direct biochemical and functional investigation. Further in vivo studies are needed to determine whether long-term topical NMN or related NAD^+^ precursors influence skin cancer development under chronic UVB exposure.

## Figures and Tables

**Table 1:** Primer sequences used for quantitative real-time PCR.

Gene	Forward Primer (5′-3′)	Reverse Primer (5′-3′)
*Gapdh*	CTACCCCCAATGTGTCCGTC	GTGTAGCCCAAGATGCCCTT
*Krt1*	GGACAACAACCGCAGTTTGG	TCTCCATGTTTGCCAGCAGT
*Krt6*	GCTGAAGCTGAGTCCTGGTA	TGCTTGGTGTTGCGTAGGTC
*Krt16*	GCAGAGCCAGGAGTACAACA	ATTCTCGCCATCCAGCAGAC
*S100a8*	GGAAATCACCATGCCCTCTAC	CACCCACTTTTATCACCATCG
*Parp1*	GCGGAGAAGACATTGGGTGA	ACCATCTTCTTGGACAGGCG
*Sirt1*	TCAGAACCACCAAAGCGGAA	CCACAGGAGACAGAAACCCC

## Data Availability

The data from the current study are not publicly available due to restricted access and institutional permission requirements. Data may be made available from the corresponding author upon reasonable request and with permission from the relevant institution.

## Supplementary Materials

The following supporting information can be downloaded at: https://www.mdpi.com/article/10.3390/ijms27219807/s1.

## Abbreviations

The following abbreviations are used in this manuscript:

NMN	Nicotinamide mononucleotide
NAD^+^	Nicotinamide adenine dinucleotide
UVB	Ultraviolet B
PARP1	Poly(ADP-ribose) polymerase 1
SIRT1	Sirtuin 1
